# Health of Cities

**Published:** 1884-09

**Authors:** 


### HALL'S JOURNAL OF HEALTH

AND

MISCELLANY.

MONTHLY.

E. K. GIBBS, A. M., M. D., EDITOR.

FOUNDED IN 1854.

### HEALTH OF CITIES.

LONGEVITY and premature decay are doubtless influenced by the food and general habits of the people, and by temperature and other local atmospheric conditions; although all these may be largely modified and brought into control by attention to sanitary laws and appliances. Artificial atmospheres are, in fact, created, in large cities, according to the character of the buildings, the air space allotted in them to each inmate, and the mode of ventilation and warming, as well as by the width of the streets, the sewerage, and other sanitary arrangements. Moreover, the hereditary constitutions of the citizens become in after generations, affected by the condition of the cities in which they and their forefathers have lived.

The facts and figures before us point to many of the causes for so great a variation in the death rate as has been shown to exist in different cities. A high death rate will, in most cases be found to be the companion of defective house accommodation, ventilation, water supply, sewerage, or scavenging. Thus, for instance, St. Petersburg, with a population of nearly a million, and the high death-rate of 35.2 per 1000, is without sewerage, and its water is taken from the river Neva, more or less contaminated by percolation from the subsoil. Cairo, with a death rate of 37 per 1000, is supplied with water from the Nile, having no sewers, and the sewerage filtering through the subsoil into the Nile, above the Water Intake of the city.

With an average of 60 people in each house (twice as many as in Paris), Vienna has a death-rate of 29.2 per 1000, while the ratable value of its houses is only one-sixth more than that of Paris. Pekin with a death-rate of 50 per 1000, is without proper sewerage, water supply, street cleansing, or other proper sanitary arrangements.

